# Estrogens in schizophrenia: What do we know?

**DOI:** 10.1192/j.eurpsy.2021.2119

**Published:** 2021-08-13

**Authors:** L. Paulino Ferreira, M. Alves, M. Duarte, A. Gamito

**Affiliations:** Psiquiatria E Saúde Mental, Centro Hospitalar de Setúbal, Setúbal, Portugal

**Keywords:** estrogens, schizophrénia

## Abstract

**Introduction:**

Schizophrenia is a chronic disease that significantly impacts cognitive functioning. Sex differences in incidence, onset and course of schizophrenia suggest estrogens have a protective role.

**Objectives:**

Our aim is to review the state of the art on this matter.

**Methods:**

Through a selection of the most relevant articles found on PubMed and ClinicalKey searching the keywords: “estrogens” and “schizophrenia”.

**Results:**

Accumulating evidence has led to the hypothesis that estrogens act as a protective factor in women regarding the onset of schizophrenia as their increase in puberty may help delay the onset of symptoms. Also, the estrogens abrupt decline in menopause may account for a second peak of onset and greater severity of the symptoms. During the menstrual cycle, when serum estrogens are at their lowest, there is an increase in the number of psychotic episodes and an exacerbation of psychotic symptoms. Pregnancy leads to an improvement of psychotic symptoms, which then worsen in postpartum. Clinical trials testing the efficacy of estrogens have been promising, which suggest they might be a useful adjuvant treatment. Despite the evidence of clinical efficacy, health risks for women using estrogen therapy should be considered, as they decrease its acceptability as a viable treatment option. The use of selective estrogen receptor modulators (SERMs), as raloxifene, could be a favorable and safer alternative.

**Conclusions:**

In conclusion, estrogens are proving to be a promising option as a complementary therapy for schizophrenia; however, further studies are needed to investigate whether they might be overall beneficial.

**Disclosure:**

No significant relationships.

